# Cost of maintaining attentional templates for multiple colors revealed by EEG decoding

**DOI:** 10.1162/imag_a_00563

**Published:** 2025-05-02

**Authors:** Melisa Menceloglu, Sari Sadiya, Susan M. Ravizza, Taosheng Liu

**Affiliations:** Department of Psychology, Michigan State University, East Lansing, MI, United States

**Keywords:** visual attention, feature, color, EEG, decoding

## Abstract

Attention to a feature enhances the sensory representation of that feature. However, it is less clear whether attentional modulation is limited when needing to attend to multiple features. Here, we studied both the behavioral and neural correlates of the attentional limit by examining the effectiveness of attentional enhancement of one versus two color features. We recorded electroencephalography (EEG) while observers completed a color-coherence detection task in which they detected a weak coherence signal, an over-representation of a target color. Before stimulus onset, we presented either one or two valid color cues. We found that, on the one-cue trials compared with the two-cue trials, observers were faster and more accurate, indicating that observers could more effectively attend to a single color at a time. Similar behavioral deficits associated with attending to multiple colors were observed in a pre-EEG practice session with one-, two-, three-, and no-cue trials. Further, we were able to decode the target color using the EEG signals measured from the posterior electrodes. Notably, we found that decoding accuracy was greater on the one-cue than on two-cue trials, indicating a stronger color signal on one-cue trials likely due to stronger attentional enhancement. Lastly, we observed a positive correlation between the decoding effect and the behavioral effect comparing one-cue and two-cue trials, suggesting that the decoded neural signals are functionally associated with behavior. Overall, these results provide behavioral and neural evidence pointing to a strong limit in the attentional enhancement of multiple features and suggest that there is a cost in maintaining multiple attentional templates in an active state.

## 1 Introduction

Visual attention is the mechanism that allows us to select relevant information from the multitude of visual inputs for prioritized processing (Chun & Wolfe, 2005;Yantis, 2000). Although attentional selection often occurs spatially, non-spatial attributes such as features and objects can also be selected (Carrasco, 2011). Feature-based attention, where selection is based on specific feature values (e.g., selecting red, selecting downward motion), has been extensively studied in the past (Liu, 2019;Scolari et al., 2014). It is generally agreed that the perceptual consequence of feature-based attention involves the enhancement of the attended features as well as the suppression of non-attended features (e.g.,Fang & Liu, 2019;Ho et al., 2012;Störmer & Alvarez, 2014;Wang et al., 2015).

To date, the vast majority of feature-based attention studies have investigated the scenario where a single feature was attended. However, in the real world, one often needs to attend to multiple features, such as looking through your closet to search for a blue shirt and a green hat or looking through your closet for either a blue or a green shirt. Both scenarios require the processing of potentially multiple features and objects, yet there are also important differences.

The first scenario is the classic divided attention task that entails the selection of two simultaneously presented objects among distractors (pants, dresses, ties), which, intuitively, is a more difficult task than selecting one object. Experimental evidence bears out this intuition, such that selecting two objects with two different features is more difficult than selecting two objects with the same feature (Lo & Holcombe, 2014;Sàenz et al., 2003), and that it is more difficult to search for multiple targets than a single target (Dombrowe et al., 2011;Stroud et al., 2011;Wolfe, 2012). The second scenario above is more concerned with the ability to prepare for the selection of multiple features among distractors (different colored shirts) in order to ultimately select one feature, and the research question is generally framed in terms of the*number of active attentional templates*, a mental representation of task-relevant information maintained in working memory (Bundesen, 1990;Desimone & Duncan, 1995;Wolfe, 1994). The general idea is that the number of simultaneously active attentional templates limits the number of objects or features that can be effectively attended. Indeed, the current study investigates this much more contentious question concerning the limits to the number of active attentional templates rather than the difficulty of dividing attention to select multiple objects.

According to an early influential theory, only one attentional template is kept active in the focus of attention, whereas the other templates are inactive, but can be maintained as accessory items (Olivers et al., 2011). This theory was able to account for results from earlier studies. However, in recent years, this single-template model has been challenged by several studies that suggest that multiple attentional templates can be simultaneously active in guiding selection (Hollingworth & Beck, 2016). Currently, there does not appear to be a consensus in the literature about this issue (reviewed inOrt & Olivers, 2020). A main contributing factor to the lack of consensus is the fairly complex behavioral paradigms used to infer the number of active attentional templates. These paradigms generally use a visual search task and often involve a secondary task such that it is difficult to equate non-perceptual factors such as decisional and strategic ones. Task complexity, thus, makes it difficult to reconcile the discrepancy in the findings between studies (see Discussion for more details).

**Fig. 1.**
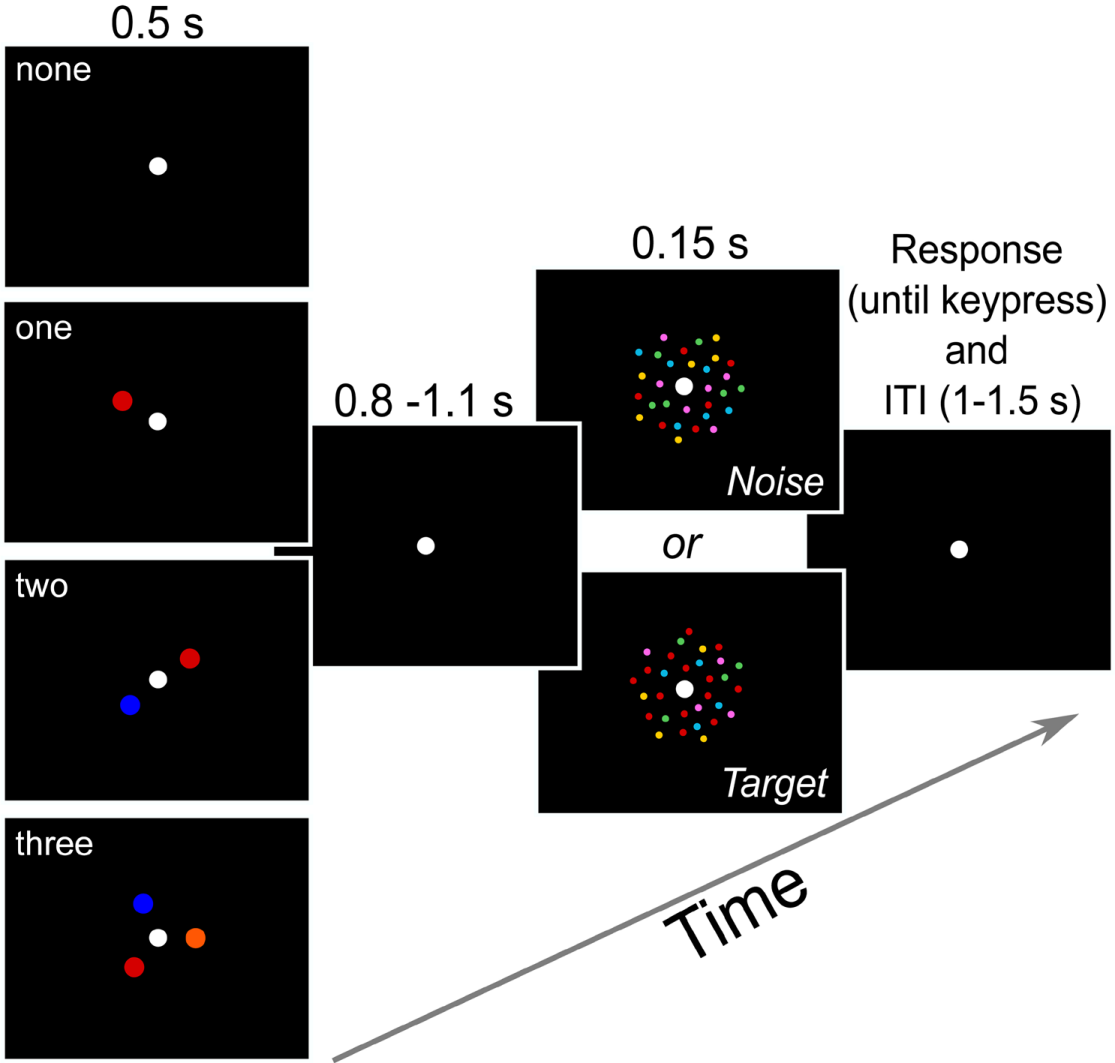
Schematic of a trial. A trial started with either no cue or one to three cues (one of the displays in the first column). The locations of the cues were randomized around the fixation point. After a brief delay, a random dot color pattern appeared, either a target pattern or a noise pattern (one of the displays in the third column). In this example, the target dot field contains an over-representation of the red color (target), and an equal proportion of the four other colors (green, yellow, cyan, and pink). The noise dot field contained an equal proportion of all five colors. For one-cue trials, the color cue always indicated the target (red), and for two- and three-cue trials, the additionally cued colors (blue and orange) never appeared in either the target or noise display. A response and inter-trial interval followed the dot display.

Here, we examined the number of active attentional templates using a novel approach. First, we used a more psychophysical design in which participants detected the presence of a color signal in noise at threshold (Fig. 1), a task employed in previous psychophysical studies (Liu & Jigo, 2017;Liu et al., 2013). Compared with visual search, this task is simpler as it does not require shifts of spatial attention or overt eye movements. Furthermore, it allows an accuracy-based measure of performance with brief stimulus exposures, which provides a complementary measure to reaction time and can be related to sensory neural mechanisms more easily (Ehrenstein & Ehrenstein, 1999). We manipulated the number of attentional templates by instructing participants to attend either one or two colors and detect a color-coherence signal.

Second, we used neurophysiological methods to more directly assess the quality of the template. Motivated by the recent success with using electroencephalography (EEG) data to decode both physically presented and remembered visual features (Bae & Luck, 2018,^2019^;Hajonides et al., 2021;Sutterer et al., 2021;Wen et al., 2022), we conducted an EEG study to investigate how attentional modulation changes with the number of attended features. Because of the higher sensitivity of multivariate analysis compared with univariate analysis, decoding provides a more direct and powerful measure of neural representations of the attended feature and allows us to examine its variation under different attentional loads. To our knowledge, only one study has examined the number of active attentional templates using neural measures.Ort et al. (2019)cued participants to prepare for either one or two colors and search for them in a subsequent stimulus array. By examining patterns of decoding accuracies, they concluded that it is possible to maintain two attentional templates although they cannot be used effectively to select two different targets. However, as we explain in more detail in the Discussion section, some design and analytical features of this study likely limit its conclusion.

Our study revealed a behavioral deficit as well as lower EEG decoding accuracy when attending to two colors versus one color. Furthermore, the neural and behavioral effects exhibited a significant correlation. These results suggest that preparing two attentional templates is not as efficient as preparing one template, thus revealing a strong limit in maintaining multiple attentional templates.

## 2 Materials and Methods

### 2.1 Participants

We tested 33 students from Michigan State University, all of whom had normal or corrected-to-normal vision and normal self-reported color vision. Three participants had low performance in the threshold task during the practice session such that accuracy could not reach the criterion level at the maximal allowed stimulus strength and hence were not tested further. Two more participants were further excluded due to noisy EEG data leading to excessive trial rejection (>40%) after EEG preprocessing. The final EEG dataset contained data from 28 participants (11 women, 16 men, 1 unreported) who were between the ages of 19 and 32 years (M = 22.31 years, SD = 3.75). Although all of the 28 participants also completed the practice session, 3 participant’s data were lost due to computer failure. Thus, the practice data contained 25 participants. We aimed to have a sample size that was near the upper limit of the sample size range from recent studies decoding EEG signals (e.g.,Bae & Luck, 2018,^2019^;Wen et al., 2022;Sutterer et al., 2021). The research protocol was approved by the university institutional review board and written informed consent was obtained from every participant.

### 2.2 Stimuli and apparatus

The experiment was controlled using MGL (Gardner et al., 2018), a MATLAB extension (The MathWorks, Natick, MA). The stimuli were presented on a black background on an LCD monitor (1920 × 1080 resolution, 120 Hz refresh rate). Participants viewed the screen at a distance of 80 cm.

The stimuli included colored disks as cues (size: 0.5°) and colored random dot fields that were composed of 240 dots (size: 0.1°) in an annulus (inner radius: 1.5°, outer radius: 6°). A white fixation dot (size: 0.3°) was present in the center of the screen throughout the experiment. The dot field was always presented in the center of the screen; the cues were presented on a virtual circle (radius: 1.5°) around the fixation point. The locations on the circle were randomized with the constraint such that multiple cues were maximally away from each other (i.e., 180° separation for two cues and 120° separation for three cues, seeFig. 1).

A total of seven possible colors were used: red, green, blue, yellow, pink, orange, and cyan; their values were set at the isoluminant level by each participant (see Procedures section for more details). There were two types of dot fields, target-absent and target-present dot fields, both containing five colors (out of the possible seven). The target-absent dot fields (noise) contained an equal number of dots for each color (48 dots per color), whereas the target-present dot fields (target) contained one color that was over-represented and the remaining four colors in equal proportions. In other words, a target is defined as a dot field containing an over-representation of a particular color. The color of the over-represented dots was the target color, and their proportion was defined as color coherence (range: 0 to 1), in analog to motion coherence in random-dot kinematograms (Britten et al., 1992). Numerically, color coherence is defined as:

Coherence=Pt=1−4Pn

*P_t_*denotes the proportion of the over-represented color, whereas*P_n_*denotes the proportion of the other non-target colors. For the noise dot fields,*P_t_*=*P_n_*= 0.2. A color-coherence threshold was measured for each participant to titrate the overall task difficulty (see Procedures section for more details).

### 2.3 Procedures

Each participant completed four procedures in a fixed order: isoluminant color setting, color-coherence threshold, behavioral practice, and EEG recording. The first three procedures occurred in a single practice session lasting ~1.5 h, and the EEG recording occurred in a separate session (usually within a week of the first session) and lasted ~2 h.

#### 2.3.1 Isoluminant color setting

In order to isolate color-based attention, we removed potential differences in perceived brightness between the colors by determining each participant’s isoluminant color values using the heterochromatic flicker photometry method (Kaiser, 1991;Lee et al., 1988). Participants viewed a flickering checkerboard pattern (8.6 Hz, check size: 1.8°) in the same annulus as the dot field stimulus. Half of the checks were drawn at a fixed gray level, whereas the other half were drawn in one of the seven possible colors. The starting value of each color was in a bright and saturated region of the RGB color space and participants adjusted the brightness of each color using the keyboard until the perceived flicker was minimized. For each color, participants performed 4 trials for a total of 28 trials, which took about 15 min. The average RGB values from the four trials were calculated to serve as the stimulus value for that color.

#### 2.3.2 Color-coherence threshold

Our task used an accuracy-based measure of attention which required us to make the task sufficiently challenging. Thus, after determining the isoluminant color values, we measured the color coherence at individual participants’ psychophysical threshold. Participants performed a single-interval color-coherence detection task, equivalent to the no-cue condition in the main task below (seeFig. 1). On each trial, after a blank interval (1.3 s), a target or a noise pattern appeared (0.15 s). Participants indicated whether they perceived a target or noise pattern by pressing one of two buttons on a keyboard. A beeping sound was played on error trials. We measured the threshold of color coherence by running seven interleaved staircases, one for each color, using a simple 2-down 1-up staircase (Levitt, 1971). A target pattern was presented on 75% of trials, and a noise pattern was presented on 25% of trials. Participants completed 5 runs of 84 trials each, with breaks between runs, in approximately 30 min. This generated 60 trials per staircase, which allowed us to estimate a color-coherence threshold for each color for each participant.

#### 2.3.3 Behavioral practice

After setting the values and coherence for each color, participants practiced the feature cueing task in the same practice session. The purpose of the practice was twofold: to familiarize participants with the behavioral task and to include more cueing conditions for a more comprehensive characterization of attentional capacity.

There were four conditions that corresponded to the number of feature cues at the beginning of each trial: no-cue, one-cue, two-cue, and three-cue (Fig. 1). The cue display (0.5 s) was followed by a fixation interval, which was fixed at 0.8 s during practice. Then either a target or noise dot field appeared for 0.15 s, followed by a response interval that ended upon response. An inter-trial interval (1.0–1.5 s) occurred after the response before the next trial started. Participants were instructed to indicate whether a target or noise dot field was presented, as accurately as possible, by pressing one of two keys on a keyboard. Furthermore, they were instructed to utilize the cued color(s) on one-, two-, and three-cue trials, to aid their task performance. Specifically, they were told to attend to the cued color(s) and detect a color-coherence signal (target, 75% trials), or the lack of such signal (noise, 25% trials). On one-cue trials, the target dot field always contained the cued color at its coherence threshold along with four other colors, whereas the noise dot field also contained the cued color but in equal proportion to all the other four colors. For two-cue and three-cue trials, one of the cued colors was over-represented in the target dot field, and the other cued colors were absent in both the target and noise dot fields (seeFig. 1caption for an example). Thus, on each trial, one of the cued colors was always present, in both the target and noise dot fields, but with different proportions. Furthermore, in the two- and three-cue trials, the non-target colors in both the target and noise dot fields did not overlap with the cued but non-target colors (seeFig. 1caption for an example). More specifically, once a target color was chosen for a trial, the other four, non-target colors were selected randomly with the constraint that the other cued but non-target color(s) could not be chosen. For instance, in a one-cue trial with a red cue (first example inFig. 1), the other four colors to be displayed were chosen at random from the pool of remaining six colors: green, blue, yellow, pink, orange, and cyan. In a two-cue trial with red and blue cues (second example inFig. 1), where red is designated as the target color and blue is designated as the cued but non-target color, the other four colors to be displayed were chosen at random from the pool of remaining five colors: green, yellow, pink, orange, and cyan. In a three-cue trial with red, blue, and orange cues (third example inFig. 1), where red is designated as the target color to be displayed and blue and orange are designated as the cued but non-target colors, then all of the four remaining colors were used in the display: green, yellow, pink, and cyan. This design reduced the possible confusion that could arise due to false matching between the cued (but non-target) color and noise color by ensuring that there was only one target color on each trial and participants must attend to the correct target color to perform the task.

The four cueing conditions were blocked and run in a pseudorandom order per participant. The blocks were organized into two superblocks, with each containing a random order of four block types for a total of 8 blocks of 84 trials each. This generated 168 trials per cueing condition, with 126 target trials and 42 noise trials. The behavioral practice took about 40 min to complete. Note that three participants’ behavioral practice data are missing as mentioned above. Thus, 25 out of 28 participants contribute to behavioral practice data while all 28 contribute to EEG data.

#### 2.3.4 EEG session

The behavioral task in the EEG session was identical to that in the practice session, with two changes. First, we only used one-cue and two-cue trials. Participants completed alternating one-cue and two-cue blocks for a total of 10 blocks of 84 trials each. Second, the cue-to-stimulus interval was jittered from 0.8 to 1.1 s, to avoid a fixed temporal phase alignment with respect to the ongoing oscillatory brain activities (e.g., alpha). We only included one-cue and two-cue conditions to obtain a sufficient number of trials for the decoding analysis. Furthermore, target dot fields were again presented on 75% of trials, to increase their number for the decoding analysis (see below for EEG data analysis). Participants completed 315 target trials (45 trials per color) and 105 noise trials (15 trials per color) for each cue condition, for a total of 840 trials. Previous studies using EEG to decode visual features have collected different amounts of data, ranging from a small number of participants with many trials (e.g., Exp 1 inSutterer et al, 2021, 5 participants, ~140 trials/condition) to a higher number of participants with fewer trials (e.g.,Bae & Luck, 2018, 16 participants, 40 trials/condition). In a recent EEG decoding study,Hajonides et al. (2021)found that both factors contribute to the ability to decode colors, and with a large number of participants (>25), even a modest number of trials (>300 trials per participant) leads to successful decoding. Based on power estimates provided byHajonides et al. (2021), our study should have an adequate level of power to decode the color signal.

### 2.4 Data analysis

#### 2.4.1 Behavioral analysis

In the practice session, participants experienced all four cueing conditions. We statistically tested the effects of cueing (one-cue vs. two-cue vs. three-cue vs. and no-cue) separately on hit rates, false alarm rates, and response times (RT) of hits and correct rejections by conducting one-way repeated measures ANOVAs and following up significant ANOVAs with planned pairwise comparisons between consecutive cueing conditions (i.e., one-cue vs. two-cue, two-cue vs. three-cue, and three-cue vs. no-cue).

In the EEG session, participants experienced only one-cue and two-cue trials. We statistically tested the effects of cueing (one-cue vs. two-cue) separately on hit rates, false alarm rates, and RT of hits and correct rejects by repeated measures t-tests. To ensure that behavioral and EEG data were completely matched, behavioral analyses only included data from trials that survived EEG-based cleaning (discussed below).

#### 2.4.2 EEG recording and preprocessing

Electroencephalography data were collected on a 64-channel BrainVision actiCHamp system (Brain Products GmbH, Gilching, Germany) at a sampling rate of 1000 Hz, referenced against AFz, using Brain Vision Recorder software. The electrodes were placed according to the 10–20 system. Eye movements were recorded using electrooculography (EOG) such that horizontal ocular activity was recorded from the outer canthi of both eyes, and vertical ocular activity was recorded from above and below the right eye. Impedances were kept near or below 10 kΩ.

The EEG and EOG data were preprocessed using the EEGLAB and ERPLAB toolboxes for MATLAB (Delorme & Makeig, 2004;Lopez-Calderon & Luck, 2014). Data were re-referenced to the average of the TP9 and TP10, which approximate the mastoids, filtered using a bandpass filter (non-causal Butterworth impulse response function, half-amplitude cutoffs at 0.01 and 80 Hz, 12 dB/octave roll-off) and an additional notch filter (Parks-McClellan notch filter) at 60 Hz to remove line noise, and down sampled to 500 Hz. Then, blink artifacts were removed via independent component analysis (ICA) using EEGLAB’s runica function (Makeig et al., 1996) such that two (*SD*= 0.82) components were removed on average. The continuous EEG data were then epoched in 3.5-s windows, time locked to the onset of the cue with a prestimulus interval of 0.5 s. After baseline subtraction (with the -0.5 to 0 s prestimulus interval as the baseline period), the epochs with artifacts were first removed using the ERPLAB’s standard algorithm that rejects epochs containing signals outside the 200-μV threshold within the -0.5 to 2 s window; the remaining epochs were visually examined for additional artifacts and further cleaned. This procedure resulted in the removal of 5.9% (*SD*= 5.8%) of the epochs on average. Additional 3.5-s epochs were created to assess the neural activity evoked by the target given the jitter between the cue onset and target onset. The activity was time locked to the onset of the target with a baseline period of -2.1 to -1.6 s (approximately the same baseline period as in the cue-locked epochs). The same artifact rejection procedure was applied.

#### 2.4.3 ERP analysis

We computed ERPs per condition and participant as the average activity over a cluster of 17 posterior electrodes (Pz, P1/2, P3/4, P5/6, P7/8, POz, PO3/4, PO7/8, Oz, O1/2), separately using cue-locked and target-locked epochs. Because our main goal was to understand how the number of attentional templates affected the neural representation of a given attended visual feature (i.e., attentional modulation of visual evoked responses), we focused on the posterior electrodes which are largely assumed to reflect visual processing. We used the same set of posterior electrodes in the ERP, spectral power, and decoding analyses. We included the entire set of posterior electrodes as similar coverage has been used in previous studies that successfully decoded color using EEG signals (Hajonides et al., 2021;Sutterer et al., 2021). We included only the target trials in all EEG analyses to match the decoding analysis (see rationale below). Nevertheless, we note that including all trials (both target and noise) does not change the general pattern of results. To evaluate the cueing effects on the ERPs at each time point, we performed cluster-based permutation tests (Maris & Oostenveld, 2007) using the MATLAB function permutest (Gerber, 2024). We used two-tailed tests with the default cluster setting of 10,000 permutations and*p*-value threshold of 0.05.

#### 2.4.4 Spectral power analysis

We analyzed event-related changes in spectral power specifically to investigate the changes in global posterior alpha during the preparatory period as a function of cueing. For each trial and for each participant, the cue-locked, artifact-free EEG waveform from each electrode was decomposed into a time series of spectral power using 60 Gabor wavelets with the parameters described in detail inMenceloglu et al. (2019). We baselined the signals using a common baseline across conditions. In particular, the time–frequency matrices from the individual trials were first averaged per condition per participant. The portion of the time–frequency matrices corresponding to the baseline period was averaged across time, averaged across trials within each condition, and then averaged across all conditions per frequency per participant. The average time–frequency matrix for each condition was divided by this common baseline and was then converted to dB (by taking the base-10 log and multiplying by 10). Resultant values were then averaged across specific electrodes and frequency bands. We focused on power in the alpha-band frequency (8–14 Hz) measured over the same 17 posterior electrodes as in the ERP analysis. We evaluated the effects of cueing on global posterior alpha power at each time point between cue onset and the end of the target presentation window by performing the same cluster-based permutation tests as discussed above.

#### 2.4.5 EEG decoding

We performed multivariate decoding analysis on EEG data using the ADAM toolbox to reveal the neural representation of color (Fahrenfort et al., 2018). We performed two sets of decoding analyses. First, we applied the decoding analysis to the cued color(s) using the cue-locked signals to reveal the neural representations of attentional templates for color. Second, the same decoding analysis was then applied to the target color using the target-locked signals to reveal the effects of attention on the neural representation of stimulus color.

We used EEG signals measured over the same 17 posterior electrodes as in the ERP and alpha-power analysis. Note that using all 62 scalp electrodes did not change the general pattern of results. We down-sampled the data to 100 Hz to speed the decoding process and applied a 15 Hz low pass filter and Gaussian smoothing with a 20-ms window on the epoched data to reduce noise (e.g.,Wen et al., 2022). We used ADAM’s default decoding method to predict conditions given observed brain activity, which implements a standard Linear Discriminant Analysis (Grootswagers et al., 2017). We used 5-fold cross-validation such that the classifier was trained on 80% of the data and tested 20% of the data (balanced across the 7 colors, randomly partitioned per participant), repeating the process until all data had been tested once. Target-color decoding was performed on target trials, as color signal (coherence) was actually present only on these trials. Note that these trials also constituted the majority of trials (75%) to ensure sufficient power to decode features. We also used target trials in the corresponding cue-color decoding as well to be able to later test the relationship between decoding and behavioral results using the same trials. Note that using both target and noise trials for cue-color decoding does not change the pattern of results.^†^When decoding cued colors in the two-cue condition, we decoded each cue color separately and averaged the results. In both decoding analyses, we repeated the procedure 10 times and averaged the classifier outputs across iterations per participant per cueing condition. We applied additional smoothing (moving mean over 40-ms window) on the resultant classifier accuracies to further reduce noise (e.g.,Bae & Luck, 2018,^2019^;Wen et al., 2022). We then performed cluster-based permutation tests as described above with a default cluster setting of 10,000 permutations and*p*-value threshold of 0.05 to identify when classifier accuracy was reliably above chance, separately for the one-cue and two-cue trials, focusing on the 1-s window post-cue or post-target. We also tested whether classifier accuracy was greater on the one-cue trials than on two-cue trials using the same method. Because there were seven possible colors to decode, the chance-level decoding accuracy was 1/7.

## 3 Results

### 3.1 Behavioral results

For all participants included, we were able to obtain stable and converging staircases for all colors. On average, participants reached a color-coherence threshold of 0.44 ± 0.01 (M ± SE) across seven colors (Red: 0.43 ± 0.02; Green: 0.41 ± 0.01; Blue: 0.39 ± 0.01; Yellow: 0.50 ± 0.02; Pink: 0.44 ± 0.01; Orange: 0.49 ± 0.02; Cyan: 0.43 ± 0.02).

For the cueing task in the practice session, participants’ performance was better on the one-cue trials than that on the two-cue trials, and on the two-cue trials than on the three-cue trials. However, performance was not different between three-cue and no-cue trials. This pattern was evident in the hit rates, false alarm rates, and response times. In particular, we found a significant cueing effect on hit rates (*F*(3,72) = 14.40,*p*< 0.001, η_p_^2^= 0.38), such that hit rates were significantly greater on the one-cue than on two-cue trials (*t*(24) = 3.48,*p*= 0.0019,*d*= 0.70) and on the two-cue than on three-cue trials (*t*(24) = 3.88,*p*< 0.001,*d*= 0.78). There were no statistical differences between three-cue and no-cue trials (*t*(24) = 0.52,*p*> 0.6,*d*= 0.10) (seeFig. 2A, left). We also found a significant cueing effect on false alarm rates (*F*(3,72) = 13.65,*p*< 0.001, η_p_^2^= 0.36), such that false alarm rates were significantly smaller on the one-cue than on two-cue trials (*t*(24) = 3.01,*p*= 0.0061,*d*= 0.60) and on the two-cue than on three-cue trials (*t*(24) = 2.75,*p*= 0.0111,*d*= 0.55). There were no statistical differences between three-cue and no-cue trials (*t*(24) = 1.01,*p*> 0.3,*d*= 0.20) (seeFig. 2A, right).

**Fig. 2.**
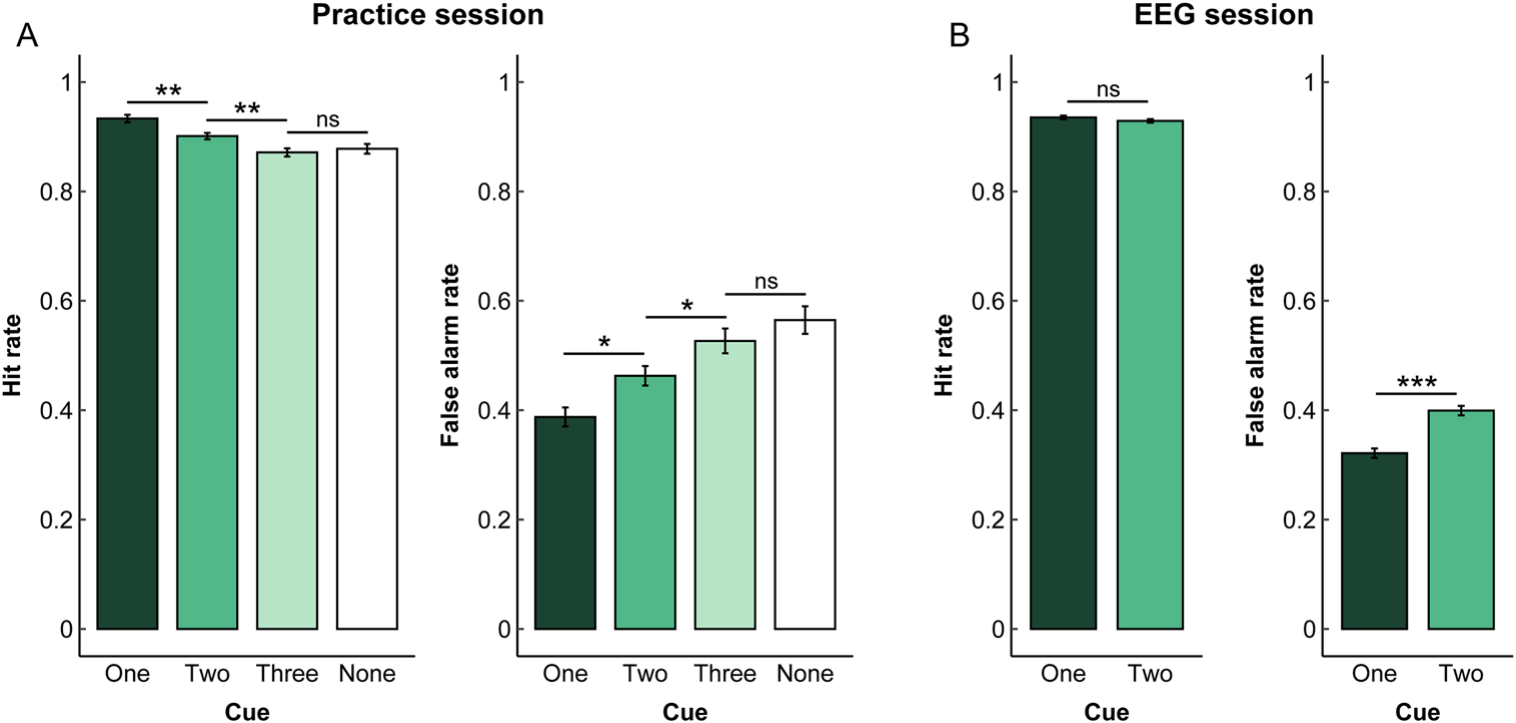
Behavioral results. (A) Hit and false alarm rates from the practice session. (B) Hit and false alarm rates from the EEG session. Error bars are ± SEM corrected for within-participant comparison (Morey, 2008). Asterisks indicate the significance level in paired t-tests, ***p< 0.0005, **p< 0.005, *p< 0.05.

As for RTs, there was a significant cueing effect on hit RTs (*F*(3,72) = 13.75,*p*< 0.0001, η_p_^2^= 0.36) as well as correct rejection RTs (RTs (*F*(3,72) = 5.91,*p*= 0.0012, η_p_^2^= 0.20). Hit RTs were significantly faster on the one-cue (489 ± 18 ms (*M*±*SE*)) than on two-cue trials (556 ± 28) (*t*(24) = 3.38,*p*= 0.0025,*d*= 0.68) and on the two-cue than on three-cue trials (625 ± 34 ms) (*t*(24) = 3.01,*p*= 0.0061,*d*= 0.60). There were no statistical differences between three-cue and no-cue trials (588 ± 26 ms) (*t*(24) = 1.49,*p*> 0.15,*d*= 0.30). Similarly, correct rejection RTs were significantly faster on the one-cue (750 ± 34 ms) than on two-cue trials (861 ± 40 ms) (*t*(24) = 3.16,*p*= 0.0042,*d*= 0.63). However, there were no statistical differences between the two-cue and three-cue trials (916 ± 59 ms) (*t*(24) = 1.25,*p*> 0.2,*d*= 0.25) or between the three-cue and no-cue trials (860 ± 46 ms) (*t*(24) = 1.33,*p*> 0.19,*d*= 0.27). RT results also confirm that there was no speed–accuracy trade off.

In the EEG session, the same participants experienced only one-cue and two-cue trials. Thus, the results were similar to the practice session, as expected. Hit rates were numerically greater on the one-cue trials than on two-cue trials but the difference did not reach statistical significance (*t*(27) = 1.37,*p*= 0.1815,*d*= 0.26) (seeFig. 2B, left). False alarms were significantly lower on the one-cue trials than on two-cue trials (*t*(27) = 6.36,*p*< 0.001,*d*= 1.20) (seeFig. 2B, right). Further, hit RT (*t*(27) = 4.06,*p*< 0.001,*d*= 0.77) as well as correct rejection RT (*t*(27) = 4.65,*p*< 0.001,*d*= 0.88) was faster on the one-cue (Hit RT: 621 ± 22, Correct Rejection RT: 852 ± 30) than on two-cue trials (Hit RT: 662 ± 24, Correct Rejection RT: 956 ± 43). We also analyzed accuracy data using signal detection analysis and obtained consistent results. Because the neural decoding focused on target trials, we present the behavioral results in hit and false alarm rates here but provide results from the signal detection analysis in Supplemental Materials section.

We note that although the hit rate overall was fairly high (~90%), false alarm rates were also high (~40%), making the task still quite difficult. We presented the target on 75% of trials to increase the number of trials for EEG decoding. This uneven target prevalence might have contributed to the relatively high hit rate. However, the overall behavioral data pattern including both accuracy and RT, and across both practice and EEG sessions, provided support for the diminished effectiveness of multiple attentional templates with an observable limit at three items in our paradigm.

### 3.2 EEG results: univariate analysis

**Fig. 3.**
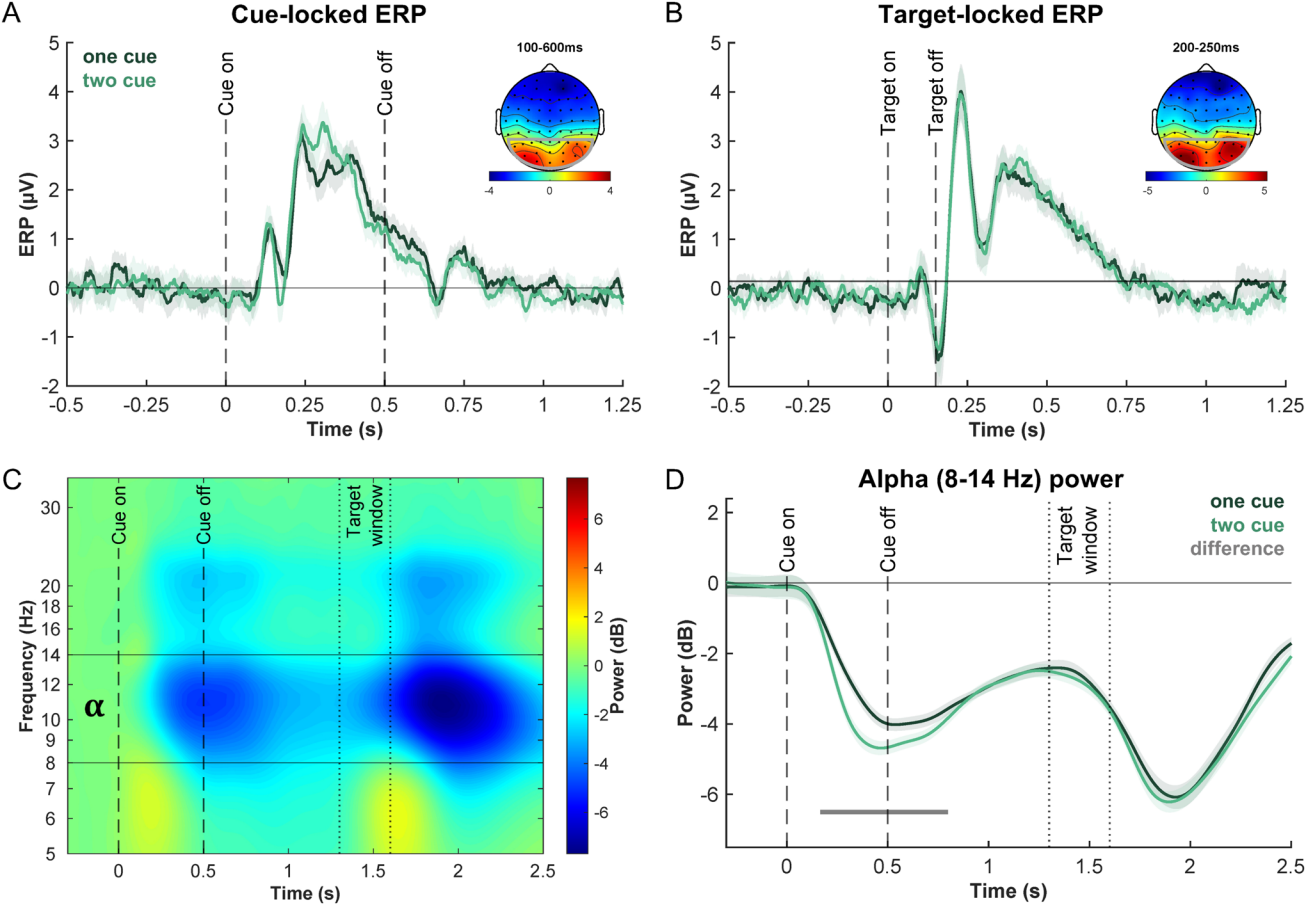
ERP and alpha-power results. (A) Cue-locked ERPs measured over the posterior electrodes, plotted for one-cue (dark green) and two-cue (light green) trials. Topography map shows the time-averaged amplitudes (100–600 ms post-cue) across all trials. (B) Same for target-locked ERPs. Topography map shows the time-averaged amplitudes (200–250 ms post-target) across all trials. (C) Time–frequency plot showing baselined power measured over the same posterior electrodes. Alpha band is specified as 8–14 Hz based on the aggregate data (shown between horizontal lines). (D) Alpha power (power averaged over 8–14 Hz) over time is plotted separately for one-cue (dark green) and two-cue trials (light green). Gray horizontal lines in line plots represent significant clusters for the cueing effect. Error bands represent ± SEM corrected for within-participant comparison.

We present the ERP findings inFigure 3A and B. Comparing the cue-locked ERPs measured over the posterior electrodes, we do not find reliable differences between one-cue and two-cue trials based on the cluster-based permutation analysis on the amplitudes (Fig. 3A). Nevertheless, the two conditions visually differ in latency. Given the unbalanced visual displays in the one-cue and two-cue trials (single colored disk vs. two colored disks present in the display) and the shape of the difference suggesting latency effects starting with the earliest visible peak, the difference likely reflects a sensory effect more so than an attentional effect. No reliable differences were observed later in the preparatory period which had the same visual display (fixation only) in both cueing conditions. Comparing the target-locked ERPs measured over the posterior electrodes, one might expect to observe visual evoked potentials with greater amplitudes in trials with stronger attentional enhancement. However, we found no reliable differences between one-cue and two-cue trials (Fig. 3B). This indicates that the attentional effect in our paradigm could not be reliably measured using the univariate signals. We note that the general pattern of results remains the same when using the more typical PO7/8 to measure early visual ERP components.

In addition to ERPs, alpha-band oscillation in EEG signals has also been associated with attention and working memory-related processes (Woodman et al., 2021). In particular, a number of studies have demonstrated that delay-period posterior alpha power, either measuring global or lateralized alpha, could track the number of items in working memory (e.g.,Erickson et al., 2017;Fukuda et al., 2015,^2016^;Heinz & Johnson, 2017). Thus, we also performed spectral power analysis and the examined potential cueing effect in alpha power.Figure 3Cshows total power measured over the posterior electrodes that combine all trials (same time locking asFig. 3A). A typical alpha reduction is observed throughout the trial compared with the baseline, reflecting general visual processing (Woodman et al., 2021). More specifically, when we examined alpha power separately for one-cue and two-cue trials (Fig. 3D), we found that the conditions differ starting at ~170 ms and lasting until ~800 ms post-cue onset. This window corresponds to cue-stimulus processing given the temporal smearing and timing of the two dips (following the cue and the dot-field onsets). This difference likely reflects differences in sensory processing (single vs. two colored disks), as the magnitude of posterior alpha suppression following stimulus onset has been linked to the amount of visual information presented (e.g.,Wang et al., 2021). Notably, we did not observe a reliable difference between the cueing conditions in the preparatory period, unlike studies manipulating working memory load (e.g.,Erickson et al., 2017;Fukuda et al., 2015,^2016^;Heinz & Johnson, 2017). This lack of sustained difference in the preparatory period may point to a lack of difference in working memory load between the cueing conditions, further suggesting a neural difference between preparatory attention and working memory.

### 3.3 EEG results: multivariate analysis

**Fig. 4.**
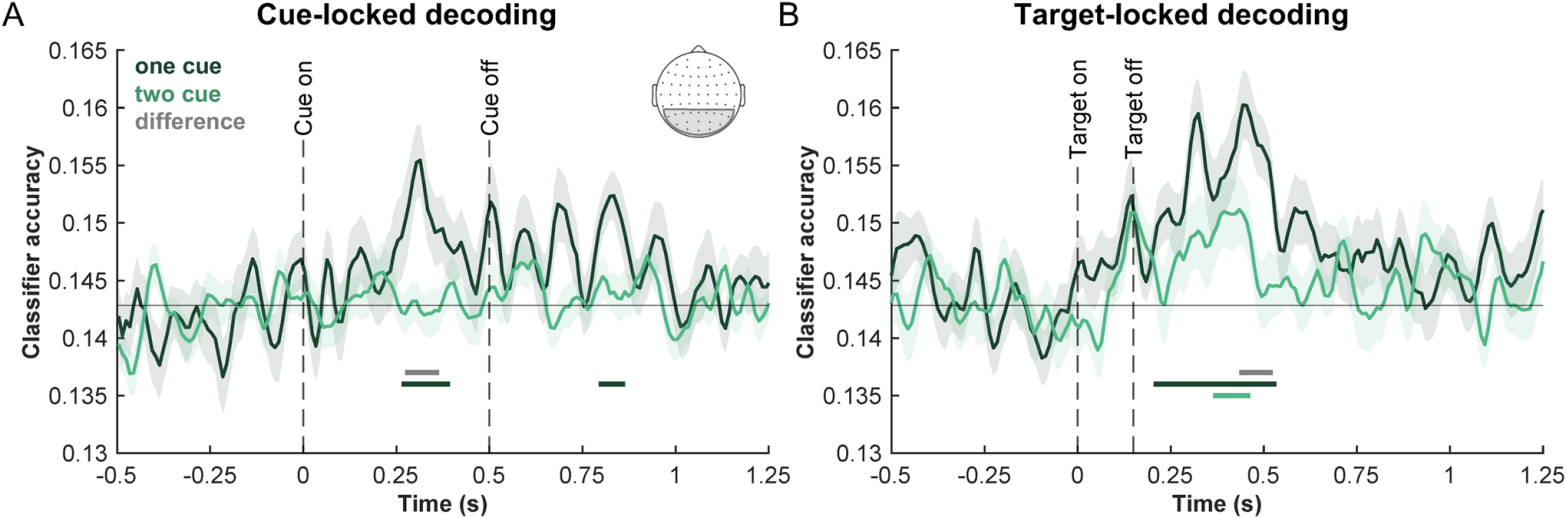
Decoding results. (A) Decoding cue color using cue-locked signals. Classifier accuracy over time is plotted separately for one-cue (dark green) and two-cue (light green) trials. (B) Decoding target color using target-locked signals. Classifier accuracy over time is plotted separately for one-cue (dark green) and two-cue (light green) trials. Horizontal lines represent significant clusters of above-chance decoding of color (>1/7) in one-cue trials (dark green), two-cue trials (light green), and the difference in classifier accuracy (one-cue > two-cue classifier accuracy, gray line). The electrodes that are used for both decoding analyses are indicated on the electrode map (gray sector). Error bands represent ± SEM corrected for within-participant comparison.

InFigure 4, we present the decoding findings. The multivariate analyses revealed reliable cueing effects on the neural representation of color both in the post-cue and post-target analysis windows. Using the cue-locked signals, we were able to reliably decode the cued color only using the one-cue trials (Fig. 4A). The classifier accuracy was significantly above chance for one-cue trials between ~260 and 390 ms post-cue, while cues were on display. Further, classifier accuracy was significantly greater for the one-cue than for two-cue trials during this window (between ~270 and 360 ms), suggesting that the cue color signal was stronger during the cue presentation on the one-cue than on two-cue trials. The classifier accuracy was also significantly above chance between ~790 and 860 ms post-cue, during the preparatory period in which the cues were removed from display. However, the classifier accuracy was not significantly different between the cueing conditions during this window.

Using the target-locked signals, we were able to reliably decode target color both for the one-cue and two-cue trials and, furthermore, we observed a reliable difference between two cueing conditions (Fig. 4B). The classifier accuracy was significantly above chance between ~200 and 530 ms for the one-cue trials and between ~360 and 460 ms for the two-cue trials. Notably, the classifier accuracy was significantly greater for the one-cue than for two-cue trials between ~430 and 520 ms post-target. In other words, the target color signal was stronger on the one-cue than on two-cue trials. Such a difference is likely due to stronger attentional enhancement of target color on the one-cue than on two-cue trials. This provides neural support for the notion that participants could more effectively attend a single color at a time.

### 3.4 Brain–behavior correlation

**Fig. 5.**
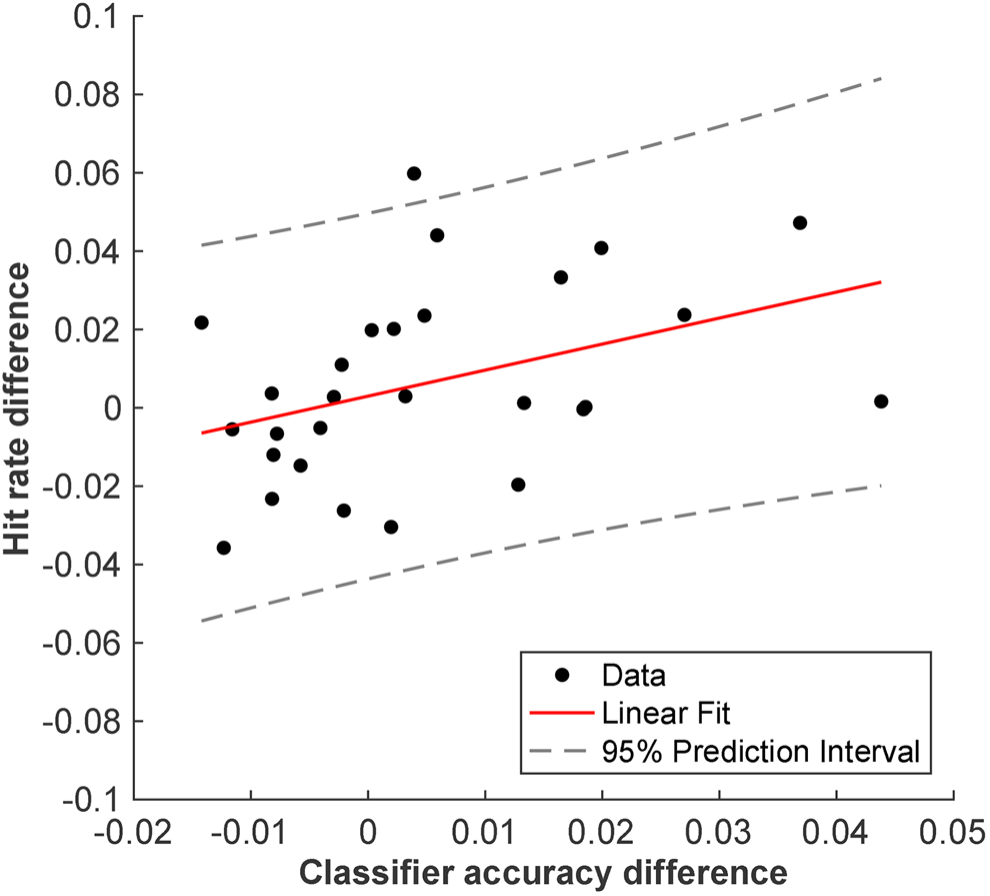
Scatterplot visualizing the correlation between the hit rate difference and classifier accuracy difference (decoding target color during stimulus period) between one-cue and two-cue trials.

Lastly, we explored potential relationships between the behavioral and neural cueing effects. Because we focused on neural decoding of the target color on target trials, we restricted our behavioral effects to these trials as well. For each participant, we obtained the behavioral cueing effect by taking the hit rate difference between one-cue and two-cue trials, and the neural cueing effect, defined by the difference in classifier accuracy between one-cue and two-cue trials. The neural cueing effect was calculated separately for the preparatory and stimulus periods, averaged across a window in each period (preparation: 0.5 to 1.25 s post-cue onset; stimulus: target onset to individual participants’ median RT per condition, 557 ms in one-cue trials, and 595 ms in two-cue trials). While the correlation between the behavioral cueing effect and the preparatory-period neural cueing effect was not reliable (*r*(27) = 0.12,*p*> 0.5), we observed a significant positive correlation between the behavioral cueing effect and the stimulus period neural cueing effect (Fig. 5,*r*(27) = 0.40,*p*= 0.0347). Thus, participants who exhibited a greater target-color decoding difference between one- and two-cue trials during the stimulus period tend to have a greater hit rate difference between these conditions. The weaker correlation between cueing effects in the preparatory activity and behavior could be due to the overall lower level of decoding during preparation and the fact that preparatory activity is further removed from the stimulus processing that informs the ultimate decision. The reliable brain–behavior correlation in the stimulus period suggests that the decoded neural signals are functionally related to behavior.

## 4 Discussion

In this study, we manipulated the number of to-be-attended features and examined its behavioral and neural consequences in a paradigm that required the selection of only a single feature. This isolates the effects of processes involved in setting up and maintaining attentional templates, rather than dividing attention between multiple presented stimuli. Our results show that as the number of templates increased, both behavioral performance and neural decoding degraded. Furthermore, the behavioral and neural effects correlated at the group level. Our results thus suggest that there is a strong limit in maintaining multiple attentional templates. Indeed, maintaining two templates incurs a cost at both the behavioral and neural levels compared with maintaining one template. In other words, to the extent that the brain can keep two templates, they are most likely kept at a reduced strength compared with a single template.

This limit can be seen in data from both the practice and EEG sessions. Behavioral data in the practice session showed a systematic performance decline as the number of templates increased from one to three. Interestingly, performance for the three-cue condition was statistically indistinguishable from that in the no-cue condition, suggesting a complete inability to use three cues to guide selection. Given that it is generally accepted that working memory storage capacity for simple features is around 3–4 items (Cowan, 2001), this finding highlights a potential dissociation between preparatory attention, which appeared to be limited to one or two templates in our data, and working memory storage. These results are broadly consistent with the notion that only items in the focus of attention, which are a subset of items in working memory storage, can be effectively used in guiding attention (McElree, 2001;Oberauer, 2002;Olivers et al., 2011). The lack of modulation in alpha power in the preparatory period also hints at a dissociation between preparatory attention and working memory. For the EEG session, we only included one-cue and two-cue conditions, due to the need to obtain sufficient trials per color to perform the decoding analysis. Behavioral data in the EEG session exhibited the same pattern as that in the practice session—better performance for one-cue than for two-cue trials. The performance difference between the conditions was weaker in the EEG than in the practice session, which might be due to learning effects from the relatively large number of practice trials (>600).

These behavioral-level effects were further corroborated by the EEG data. By examining the multivariate voltage pattern over the scalp, we were able to reliably decode the color of the target for both the one-cue and two-cue conditions. This result is in agreement with recent demonstrations of using EEG to decode physically presented visual features (Bae & Luck, 2019;Sutterer et al., 2021). However, we note that decoding in our task is likely more difficult as our target was a weak, threshold-level color-coherence signal; thus, the relatively low decoding accuracy we observed may be more consistent with the decoding of remembered features (Bae & Luck, 2018;Wen et al., 2022). Importantly, however, we found a reliable difference in EEG decoding accuracy between the two cueing conditions. Compared with the two-cue condition, decoding accuracy was both more sustained and more elevated in the one-cue condition. Furthermore, the cueing effect measured by behavioral performance and decoding accuracy exhibited a positive correlation. These results provide a neural basis for the reduced behavioral cueing effect as the number of attentional templates increases and implies a less discriminatory neural representation as the underlying cause.

There has been intense research in recent years on the number of attentional templates that can be simultaneously active. Most of this research has employed behavioral methods involving the visual search paradigm. A single-template model was initially proposed to explain the pattern of effects from attentional guidance on search behavior (Olivers et al., 2011), however, more recent work has challenged this idea and, instead, has provided evidence supporting the multiple-template model. A major obstacle in this area is the indirect, and often complicated methods used to infer the status of the attentional template, which makes it difficult to control strategic and task-related factors. For example, in one set of experiments, participants made multiple eye movements to search for one of two color targets. Template status was inferred by the switch cost in fixation duration and saccade latency when participants looked at different colors between successive fixations (Beck et al., 2012;Ort et al., 2017). These studies found that the switch cost can either appear or disappear depending on the availability of the second target, suggesting the possibility of proactively switching before the critical saccade. Another series of studies used the memory-based attentional capture paradigm while manipulating the number of to-be-remembered items (Bahle et al., 2018;Chen & Du, 2017;Hollingworth & Beck, 2016;van Moorselaar et al., 2014). This paradigm contains two tasks which have many parameters that could contribute to the discrepant findings. Indeed, a recent study using two very similar memory-based attentional capture tasks yielded inconsistent results that supported the single- and multiple-template model, respectively, without an obvious resolution (Frătescu et al., 2019). While more work is needed to improve these behavioral methods as well as to assess how different experimental factors contribute to results, our paradigm offers a more straightforward measure of the efficacy of the attentional template due to its simplicity: participants actively used one or two templates to detect a threshold signal in a single-interval task at a single location, eliminating the need for overt eye movement, stimulus and task switching, and prioritization between tasks.

Although the literature is dominated by behavioral studies, one recent study used EEG decoding to measure the number of attentional templates and arrived at a different conclusion than ours (Ort et al., 2019). The authors varied the number of color cues (one-cue vs. two-cue) and instructed participants to search for two objects, either of the same color (one-color) or two different colors (two-color), in a search array of heterogeneous colors. When they compared the one-cue/one-color with two-cue/one-color condition, which is analogous to our comparison, they only found minimal behavioral and decoding differences. When they compared the two-cue/one-color with two-cue/two-color condition, however, a much larger deficit was observed for both behavioral performance and decoding accuracy. They conclude that humans can maintain two attentional templates allowing them to effectively select an object with either feature, but cannot use them effectively to select two different objects. In contrast, our comparison between the one-cue and two-cue conditions revealed a more substantial difference than Ort’s study, as they only observed a slight delay in decoding without any difference in the asymptote. There are at least two factors that might contribute to this discrepancy. First, the target objects in their study always appeared on the cardinal axes, which could facilitate perceptual grouping between the two objects when they were of the same color. This grouping opportunity was present in the one-cue/one-color and two-cue/one-color condition but was unavailable in the two-cue/two-color condition, which could diminish the difference between the first two conditions and accentuate the difference between them and the third condition. Second, we note thatOrt et al. (2019)decoded the*location*of the two target objects, instead of their colors. Thus, their decoding results are an indirect measure of feature selection which could be influenced by other factors such as perceptual grouping. We believe that these two factors might have contributed to an underestimation of the impact of maintaining two attentional templates. More work is needed to tease apart how these methodological differences contributed to observed results.

Given the general notion that attentional templates are maintained in working memory (Bundesen, 1990;Desimone & Duncan, 1995;Wolfe, 1994), and the fact that remembered visual features can be decoded from the delay period activity of a working memory task (D’Esposito & Postle, 2015), one might expect that we should be able to decode the cued color during the preparatory period, that is, after the cue and before the stimulus. Indeed, we observed a higher decoding accuracy for one-cue than for two-cue condition during both the cue presentation and the subsequent preparatory period (Fig. 3C). This difference was statistically reliable during the cue period, presumably reflecting a sensory instead of an attentional effect. The difference in the preparatory period likely reflects an effect due to the maintenance of the attentional template and is consistent with the idea that a single template is maintained at a higher fidelity than two templates. However, the numeric advantage for the one-cue condition did not reach statistical significance, and thus does not warrant strong conclusions. Overall, we interpret our preparatory period finding as a subtle effect that requires more sensitive methods or more statistical power to establish. Importantly, however, the stimulus period can be used to determine the efficacy of applying the attentional templates, because the selection demand was equated between the two conditions (both involved the selection of a single target). Thus, the observed decoding difference in the stimulus period can be used to infer the status of the attentional template and support the idea that keeping multiple attentional templates active comes at a cost. We do acknowledge that our effects are generally somewhat subtle, which may be improved by comparing conditions with a larger behavioral difference (e.g., one-cue vs. three-cue). Future studies are needed to optimize the detection of attentional templates during the preparatory period and potentially extend to higher set-sizes. Another potentially interesting future direction is to explore functional connectivity between frontal and posterior electrodes and relate it to number of attentional templates (Chiarion et al., 2023).

Our results inform the underlying mechanisms regarding the number of attentional templates that can be actively maintained. In general, they argue against a strong version of the multiple-template model, where two templates can each be maintained at the same level of strength as a single template. However, these results are compatible with a weakened version of the multiple-template model where two templates can be maintained but each at a reduced strength. Alternatively, a single-template model that incorporates switching between two templates could also potentially account for our results. Under this model, participants can only attend to one feature at a time but can switch between two features given the opportunity. Thus, on two-cue trials, participants would sometimes attend to the cued target color, leading to a strong attentional effect, and sometimes they would attend to the other cued but non-presented color, resulting in no attentional effect. Pooling across these two types of trials will generate an intermediate level of performance, hence lower than that in the one-cue condition.

Unfortunately, these two models, a weakened two-template model and a single-template model (with switching between two templates), are very difficult to resolve. Analogous questions have been addressed when attention needs to be divided between two physically presented features. For example,Ort et al. (2019)looked for any alternation pattern in their decoding results for the two target locations but found none. However, other studies that examined attention to two features using a resetting cue found rhythmic alternation between the two features (Mo et al., 2019;Re et al., 2019). It is not clear what caused the discrepancy between these results. More importantly, with regard to attentional templates, the ultimate question is concerned with the preparatory period, that is, whether two templates are maintained simultaneously or alternatively during the preparatory period. This appears to be an even more difficult problem than dividing attention between two features, and it is also possible that different mechanisms underlie the maintenance of two templates and active selection between two features. Thus, we believe it is premature at this point to rule out either model, and more work is needed.

In summary, our findings suggest that humans cannot effectively maintain two templates as well as a single template, with a possible ceiling at three templates. At least two possible models can explain our results: either multiple templates are maintained simultaneously albeit at a weaker level, or a single template is active at a time with the possibility of rapid switching. These results do not support a model where multiple templates can be maintained without cost, thus informing the debate regarding the capacity of multiple target search. The observed cost when maintaining multiple templates also parallels the cost when multiple physically presented features need to be selected (e.g.,Lo & Holcombe, 2014;Sàenz et al., 2003), suggesting competitive interactions exist between feature representations during both stimulus selection and preparation. Future work is needed to distinguish candidate models regarding the number of active templates and to explore the connection between stimulus-based and memory-based competition.

## Supplementary Material

Supplementary Material

## Data Availability

Materials and data for the experiments reported here are available online athttps://osf.io/2tbwv/(DOI:10.17605/OSF.IO/2TBWV). The experiments were not preregistered.

## Footnotes

† In addition, we also explored decoding using noise trials. Successful decoding of the cued color on these trials may reveal illusory percept of a color signal. However, we did not find any reliable decoding results. This may be related to the lack of a coherent sensory signal and low trial count.
