# Supplementary material for: Cost of maintaining attentional templates for multiple colors revealed by EEG decoding
Source: Imaging Neurosci (Camb). 2025 May 2;3:imag_a_00563. doi: 10.1162/imag_a_00563 (PMC12319952; doi:10.1162/imag_a_00563)
Supplement: Supplementary Material [file imag_a_00563-supp.pdf]

Supplemental Material

Here, we present the results of the signal detection analysis, reporting both d-prime and response criterion for the practice session and EEG session in **Figure S1**.

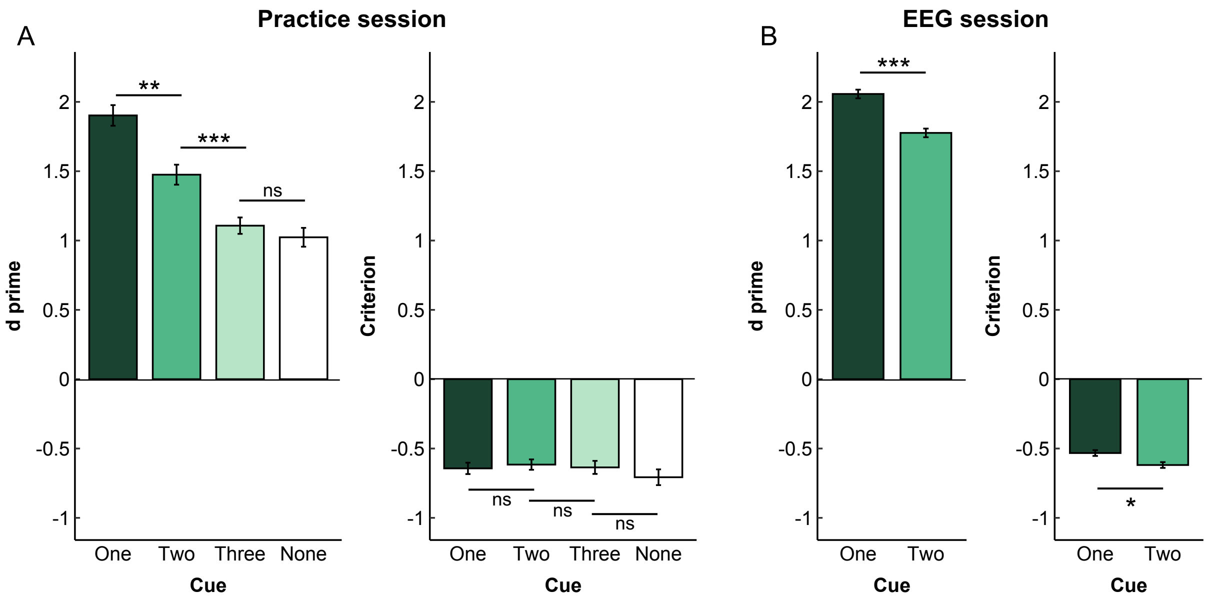

Figure S1. Signal detection analysis results. A) d prime and criterion for the practice session. B) Same for the EEG session. Error bars are ±SEM corrected for within-participant comparison (Morey, 2008). Asterisks indicate the significance level in paired t-tests, ***p < .0005, **p < .005, *p < .05.

In the practice session, in which the participants experienced one-, two-, three- and no-cue conditions, we found a significant cueing effect on sensitivity ($F(3,72) = 34.16$, $p < .0001$, $\eta_p^2 = 0.59$) such that sensitivity was greater on the one-cue trials (M ± SE: 1.90 ± 0.13) than two-cue trials (1.47 ± 0.15) ($t(24) = 3.81$, $p = .0009$, $d = 0.76$) and on the two-cue trials than three-cue trials (1.10 ± 0.11) ($t(24) = 4.08$, $p = .\ 0004$, $d = 0.82$). There were no statistical differences between three-cue and no-cue trials (1.02 ± 0.11) ($t(24) = 0.90$, $p > .3$, $d = 0.18$). While participants had liberal decision criterion in all conditions (-0.65 ± 0.04), the bias was not statistically different between conditions ($F(3,72) = 0.73$, $p > .5$, $\eta_p^2 = 0.03$) (**Fig. S1 A**).

In the EEG session, in which participants experienced one- and two-cue conditions, we also found a significant effect of cueing on sensitivity such that sensitivity was greater on the one-cue (2.06 ± 0.10) than two-cue trials (1.78 ± 0.10) ($t(27) = 6.36$, $p < .0001$, $d = 1.20$). While participants had liberal decision criterion in both conditions, criterion was less liberal on the one-cue (-0.53 ± 0.03) than two-cue trials (-0.62 ± 0.04) ($t(27) = 2.92$, $p = .0070$, $d = 0.55$). This small but reliable difference in criterion between one- and two-cue trials suggests that

participants were on average more liberal on two-cue trials (**Fig. S1 B**). We note that this effect is numerically smaller than the effect in sensitivity. Furthermore and importantly, our neural analysis focused on decoding the visual features, which should be orthogonal to overall response bias. We also examined potential relationships between cueing effects in response bias measures (criterion, false alarms) and neural decoding difference, similar to the analysis in the main text, and found no significant correlations (all *p's* >.1). Lastly, we note that this effect was not reliably observed as it was absent in the practice session. Thus, we believe that while there is a small criterion change between the one- and two-cue conditions in the EEG session, it does not impact the overall interpretation of the main results.
